# 非小细胞肺癌脑转移患者接受一代表皮生长因子受体酪氨酸激酶抑制剂治疗的临床研究

**DOI:** 10.3779/j.issn.1009-3419.2017.02.06

**Published:** 2017-02-20

**Authors:** 惠幸 董, 少华 崔, 峰 潘, 莉莉 董, 艳洁 牛, 怡卓 赵, 爱琴 顾, 晓燕 金, 丽岩 姜

**Affiliations:** 1 200030 上海，上海交通大学附属胸科医院呼吸内科 Department of Respiratory Medicine, Shanghai Chest Hospital, Shanghai Jiao Tong University, Shanghai 200030, China; 2 200336 上海，上海交通大学医学院附属同仁医院呼吸内科 Department of Respiratory Medicine, Tongren Hospital Shanghai Jiao Tong University School of Medicine, Shanghai 200336, China

**Keywords:** 肺肿瘤, 脑转移, 表皮生长因子受体-酪氨酸激酶抑制剂, 生存期, Lung neoplasms, Brain metastases, Epidermal growth factor receptor-tyrosine kinase inhibitors, Survival

## Abstract

**背景与目的:**

非小细胞肺癌（non-small cell lung cancer, NSCLC）脑转移患者接受一代表皮生长因子受体酪氨酸激酶抑制剂（epidermal growth factor receptor-tyrosine kinase inhibitors, EGFR-TKIs）的生存数据及影响因素未完全阐明。本研究对存在脑转移的NSCLC患者的生存数据进行分析，以期为指导临床实践提供一定的研究证据。

**方法:**

回顾性收集上海交通大学附属胸科医院2012年-2013年确诊肺癌脑转移并接受一代EGFRTKIs治疗的病例。采用*Kaplan*-*Meier*单因素、*Cox*多因素分析方法，探讨NSCLC脑转移患者接受EGFR-TKIs的生存情况及影响因素。

**结果:**

总体人群中位无进展生存时间（progression-free survival, PFS）为10.0个月（95%CI: 8.3-11.7），中位生存时间（overall survival, OS）为28.0个月（95%CI: 22.9-33.1）。病理组织类型、肿瘤分化程度分别是患者接受EGFR-TKIs后PFS、OS的独立影响因素（*P*分别为0.001、0.050）。

**结论:**

NSCLC脑转移患者接受一代EGFR-TKIs具有良好的疗效，腺癌亚型患者的PFS长于非腺癌患者，其他肿瘤分化程度患者的OS长于肿瘤低分化患者。

## Abstract (en)

**Background and objective:**

A survival analysis and the influencing factors for non-small cell lung cancer (NSCLC) patients with brain metastases accepting first-generation epidermal growth factor receptor tyrosine kinase inhibitor (EGFR-TKIs) treatment have not yet been elucidated to date. In this study, we collected and analyzed the survival data of NSCLC patients with brain metastasis to obtain evidence and to provide guidance in clinical practice.

**Methods:**

NSCLC patients with brain metastases who were treated with first-generation EGFR-TKIs were retrospectively collected in 2012-2013 from Shanghai Chest Hospital, Shanghai Jiao Tong University. The *Kaplan*-*Meier* method and *Cox* regression were performed for univariate and multivariate analyses, respectively, to explore the independent predictors influencing the survival of patients with NSCLC brain metastases.

**Results:**

The median progression-free survival (PFS) and median overall survival (OS) of all patients treated with first-generation EGFR-TKIs were 10.0 months (95%CI: 8.3-11.7) and 28.0 months (95%CI: 22.9-33.1), respectively. Pathological subtypes were the independent predictors of PFS (*P*=0.001), and tumor differentiations were the independent predictors of OS (*P*=0.050).

**Conclusion:**

First-generation EGFR-TKIs showed promising efficacy in NSCLC patients with brain metastases. PFS was longer in patients with adenocarcinoma than in those with a non-adenocarcinoma subtype. OS was longer in patients with differentiated tumors than in those who developed poorly differentiated tumors.

脑部是非小细胞肺癌（non-small cell lung cancer, NSCLC）转移最容易累及的器官，约10%-20%的NSCLC患者在就诊时就发现发生了脑转移^[1, 2]^，还有约20%的NSCLC患者在发现原发灶之后若干时间后被发现脑转移^[2, 3]^。合并脑转移的NSCLC预后差，即使接受过规范化放疗的患者，其中位生存期（median overall survival, mos）也只有8.8个月^[3]^。

目前对于表皮生长因子受体（epidermal growth factor receptor, *EGFR*）突变阳性的NSCLC患者，EGFR-酪氨酸激酶抑制剂（EGFR-tyrosine kinase inhibitors, EGFR-TKIs）已成为一线治疗手段。第一代EGFR-TKIs的代表药物包括吉非替尼、厄洛替尼、埃克替尼等，因其作用更精准，对存在*EGFR*敏感突变的NSCLC，具有良好的疗效和可接受的毒副反应，患者应用后生活质量也较传统化疗有明显提高^[4, 5]^。然而，由于标准剂量下TKI在脑脊液中浓度不足，有效率低^[6]^，对于脑转移病灶，特别是存在症状性脑转移的患者，是否将EGFR-TKIs作为一线治疗手段仍存在争议^[7]^。

目前针对脑转移患者的相关研究^[8, 9]^显示一代EGFR-TKI可以控制EGFR突变阳性患者的脑转移病灶的进展。其他一些研究^[10-12]^也提示*EGFR*突变阳性合并脑转移的患者接受EGFR-TKI治疗联合脑部放疗较单纯放疗效果更好。但EGFR-TKI应用于脑转移显示出疗效的同时，也存在着一些困惑。部分患者在用药期间，肺部病灶稳定，但却发生了脑转移或原有脑转移病灶治疗有效后再次进展。Omuro等^[7]^提出单独采用EGFR-TKI治疗NSCLC脑转移的颅内复发率较高，而且无进展时间较短。

以上论述表明，脑转移NSCLC患者接受一代EGFRTKIs的生存数据，以及影响生存的因素并未完全阐明。因此，本研究拟在存在*EGFR*敏感突变合并脑转移的人群中研究接受一代EGFR-TKIs治疗的生存情况，并探索影响生存的因素。

## 1 材料与方法

### 1.1 研究资料

收集2012年1月1日-2013年12月31日在上海交通大学附属胸科医院经病理学或细胞学检查确诊NSCLC伴有脑转移的患者作为研究对象。具体入排如下：入选标准：①患者年龄大于18岁；②经组织病理学或细胞学检查确诊为NSCLC；③有详细的头颅计算机断层扫描（computed tomography, CT）或头颅增强磁共振成像（magnetic resonance imaging, MRI）资料确诊肺癌伴有脑转移；④有明确TNM分期；⑤接受过一代EGFR-TKIs治疗；⑥病理组织学标本经过探针扩增阻滞突变系统聚合酶链反应（ARMS-PCR）存在*EGFR*基因突变的患者。排除标准：①合并其他系统的恶性肿瘤；②排除主要观察指标数据不完整者。

对符合入组条件的患者收集其主要的临床信息，包括：性别、年龄、吸烟史、体力状况评分（Eastern Cooperative Oncology Group performance status, ECOG PS）、组织病理学类型、分化程度、脑转移病灶数、脑放疗情况、*EGFR*突变状况、EGFR-TKIs治疗情况等。

### 1.2 干预措施

符合入排标准的患者均接受第一代EGFRTKIs（吉非替尼、厄洛替尼或埃克替尼）治疗，具体用法用量为：吉非替尼250 mg，*qd*口服治疗，厄洛替尼150 mg，*qd*口服治疗，埃克替尼125 mg，*tid*口服。患者服药直至出现肿瘤进展或出现不能耐受的不良反应。若患者颅内病灶无进展则继续定期观察，若颅内病灶进展则加用颅脑放疗治疗。全部患者开始治疗后1个月复查肺部增强CT，脑部MRI，腹部超声和骨扫描以评价。随访期间每2个月定期复查肿瘤相关影像学检查直至疾病进展。

### 1.3 观察指标及随访方法

①主要观察指标是PFS和OS。PFS定义为患者开始接受EGFR-TKIs治疗至出现疾病进展的时间（包括出现颅内进展或者颅外病灶进展）。OS定义为入组患者开始接受EGFR-TKIs治疗至死亡的时间。本研究的研究节点为2016年10月31日；②疾病进展数据：疗效采用实体瘤疗效评估标准1.1版本（Response Evaluation Criteria in Solid Tumors 1.1, RECIST 1.1）进行评估。疾病进展（progressive disease, PD）：以整个实验研究过程中所有测量的靶病灶直径之和的最小值为参照，直径和相对增加至少20%（如果基线测量值最小就以基线值为参照）；除此之外，必须满足直径和的绝对值增加至少5 mm（出现一个或多个新病灶也视为疾病进展）；③生存数据：OS数据采用电话方式对所有患者进行随访并记录。

### 1.4 统计学方法

应用SPSS 17.0软件进行统计学分析。计数资料采用“例数（构成比）”的方式表示。采用*Pearson*卡方检验或*Fisher*确切概率法（当不符合*Pearson*卡方检验应用条件时采用*Fisher*确切概率法）分析存在19del和21L858R两组突变患者间的临床特征。采用*Kaplan*-*Meier*法进行单因素生存分析，*Log*-*rank*法（pooled over strata）进行单因素两水平比较。采用*Cox*比例风险模型进行多因素生存分析（*Enter*法，因素选入标准为0.05，排除标准为0.1）。对于失访数据，如是在用药有效后半年内失访则不纳入最终分析，超过半年后失访的数据按照截尾数据进行处理。本研究所用统计推断均为双侧检验，*P* < 0.05为差异有统计学意义。

## 2 结果

### 2.1 一般资料

2012年1月1日-2013年12月31日共有201例患者接受EGFR-TKIs治疗。本研究最终共有138例符合具体的入排标准。患者的一般临床资料列于表 1。本研究人群大多为不吸烟、女性、腺癌亚型，患者确诊时的临床分期集中在Ⅲ期（52.8%）和Ⅳ期（11.4%），确诊时已发生脑转移的患者有43例（31.2%）。入组时患者均发生脑转移，都已经进展至Ⅳ期，多存在*EGFR*常见的19del和21L858R突变，接受吉非替尼、厄洛替尼和埃克替尼的人数分别是58例、47例和33例，接受第一代EGFR-TKIs治疗的时机集中在一线和二线。

**1:** 138例患者的人口学和临床病理特征 Demographic and clinicopathologic characteristics of the patients (*n*=138)

Characteristic		*n* (%)
Age (yr)	< 60	87 (63.0)
	≥60	51 (37.0)
Gender	Male	61 (44.2)
	Female	77 (55.8)
Smoking history	None	98 (71.0)
	Yes	40 (29.0)
ECOG PS	0	21 (15.2)
	1	117 (84.8)
Pathological type	Adenocarcinoma	130 (94.2)
	Non-adenocarcinoma	8 (5.8)
Differentiation	Low	34 (24.6)
	Other	104 (75.4)
Brain metastasis	Single site	42 (30.4)
	Multiple sites	96 (69.6)
Radiotherapy for brain metastasis	No	29 (21.0)
	WBRT	78 (56.5)
	SRS	20 (14.5)
	SRS+WBRT	11 (8.0)
EGFR status	19del	68 (49.3)
	21L858R	51 (37.0)
	Other	19 (13.7)
EGFR-TKIs	Gefitinib	58 (42.0)
	Erlotinib	47 (34.1)
	Icotinib	33 (23.9)
Treatment line	First-line	48 (34.8)
	Second-line	64 (46.4)
	Other-line	26 (19.8)
ECOG PS: Eastern Cooperative Oncology Group performance status; EGFR: epidermal growth factor receptor; TKI: tyrosine kinase inhibitor; 19del: deletions in Exon 19; 21L858R: point mutation in Exon 21; WBRT: whole brain radiotherapy; SRS: stereotaxic radiosurgery.		

### 2.2 总体人群生存数据

截止研究节点，共有131例（94.9%）患者发生PD，总体中位PFS为10.0个月（95%CI：8.3-11.7，图 1A）。电话随访共有1 4例（10.1%）失访数据，截止研究节点共有82例（59.4%）患者死亡，总体中位OS为28.0个月（95%CI：22.9-33.1，图 1B）。

**1:**
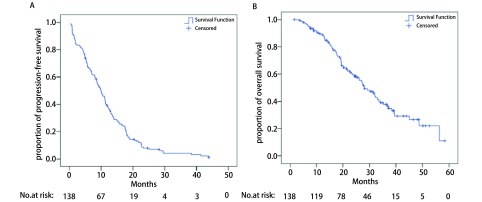
总体无进展生存时间和总生存时间曲线。A：总体无进展生存时间曲线，刻度标记代表删失数据。总体中位PFS为10.0个月（95%CI: 8.3-11.7）。B：总体生存时间曲线，刻度标记代表删失数据。总体中位OS为28.0个月（95%CI: 22.9-33.1）。 Survival curves for progression-free survival (PFS) and overall survival (OS) in all patients. A: A survival curve for progression-free survival in all patients, tick marks represent censored data. The median progression-free survival for all patients was 10.0 months (95%CI: 8.3-11.7). B: A survival curve for overall survival in all patients, tick marks represent censored data. The median overall survival for all patients was 28.0 months (95%CI: 22.9-33.1).

### 2.3 PFS影响因素分析结果

*Kaplan*-*Meier*法单因素生存分析结果显示，病理组织类型是患者接受EGFR-TKIs后PFS的影响因素[腺癌*vs*非腺癌，10.1个月（95%CI: 8.6-11.6）*vs* 1.9个月（95%CI: 1.7-2.1），*P*=0.004，图 2A]，而性别、年龄、吸烟史、ECOG PS、分化程度、脑转移病灶数、脑放疗情况、*EGFR*突变情况、EGFR-TKIs治疗时机等均不是患者接受EGFR-TKIs后PFS的影响因素。PFS的*Kaplan*-*Meier*法单因素生存分析结果详见表 2。

**2:**
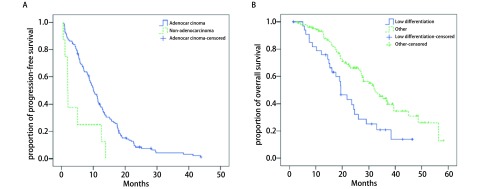
单因素生存分析相关的生存曲线。A：不同病理学类型的无进展生存时间生存曲线，刻度标记代表删失数据。腺癌患者*vs*非腺癌患者的中位无进展生存时间为10.1个月（95%CI: 8.6-11.6）*vs* 1.9个月（95%CI: 1.7-2.1），*P*=0.004。B：不同分化程度的总生存时间曲线，刻度标记代表删失数据。低分化*vs*其他分化总中位生存时间为19.5个月（95%CI: 16.0 -23.0）*vs* 32.4个月（95%CI: 26.1-38.7），*P*=0.002。 Survival curves related to univariate survival analysis. A: Progression-free survival curves for in pathological type, tick marks represent censored data. The median progression-free survival for patients with adenocarcinoma *vs* patients with non-adenocarcinoma was 10.1 months (95%CI: 8.6-11.6) *vs* 1.9 months (95%CI: 1.7-2.1), *P*=0.004. B: Overall survival curves for patients in tumor differentiation, tick marks represent censored data. The median overall survival for patients with low tumor differentiation *vs* patients with other differentiation was 19.5 months (95%CI: 16.0-23.0) *vs* 32.4 months (95%CI: 26.1-38.7), *P*=0.002.

**2:** 无进展生存时间的*Kaplan*-*Meier*法单因素生存分析 Univariate survival analysis by *Kaplan*-*Meier* method for PFS

Covariates	Comparisons	Median PFS in Months (95 % CI)	*P*
Gender	Male *vs* Female	9.2 (5.6-12.9) *vs* 10.4 (8.7-12.1)	0.248
Age (yr)	≤60 *vs* > 60	9.7 (7.5-11.9) *vs* 10.1 (7.5-12.7)	0.507
Smoking history	None *vs* Yes	10.4 (8.7-12.0) *vs* 6.8 (1.2-12.5)	0.384
ECOG PS	0 *vs* 1	11.3 (6.1-16.6) *vs* 9.8 (8.3-11.4)	0.512
Differentiation	Low *vs* Other	8.6 (7.2-9.9) *vs* 10.3 (8.7-11.9)	0.257
Pathological type	Adenocarcinoma *vs* Non-adenocarcinoma	10.1 (8.6-11.6) *vs* 1.9 (1.7-2.1)	0.004*
Brain metastasis	Single *vs* multiple	9.4 (7.7-11.1) *vs* 10.1 (8.2-12.0)	0.763
Radiotherapy	No *vs* WBRT *vs* SRS *vs* SRS+WBRT	9.0 (5.1-12.9) *vs* 9.5 (7.2-11.7) *vs* 9.8 (5.7-13.9) *vs* 15.7 (11.1-20.3)	0.706
EGFR status 1	19del *vs* 21L858R *vs* Other	11.2 (9.8-12.6) *vs* 8.9 (7.2-10.7) *vs* 6.4 (1.7-11.2)	0.799
EGFR status 2	Common *vs* Other	10.1 (8.6-11.6) *vs* 5.0 (2.5-7.6)	0.271
EGFR-TKIs	Gefitinib *vs* Erlotinib *vs* Icotinib	11.1 (9.2-13.0) *vs* 9.4 (5.9-12.9) *vs* 8.9 (6.6-11.3)	0.766
Treatment line 1	First-line *vs* Second-line *vs* Other-line	8.5 (5.2-11.8) *vs* 11.1 (9.7-12.4) *vs* 6.5 (4.6-8.5)	0.996
Treatment line 2	First-line *vs* Non-first-line	8.5 (5.2-11.8) *vs* 10.3 (8.5-12.1)	0.926
PFS: progression-free survival. ^*^P < 0.05.			

*Cox*多因素生存分析结果显示，病理组织类型是患者接受EGFR-TKIs后PFS的独立影响因素（*P*=0.001）。PFS的*Cox*多因素生存分析结果详见表 3。

**3:** 无进展生存时间的*Cox*多因素生存分析 *Cox* multivariate survival analysis for PFS

Covariates	B	SE	Wald	*P*	Exp (B)	95%CI for Exp (B)	
						Lower	Upper
Gender	0.117	0.242	0.233	0.629	1.124	0.699	1.806
Age	-0.037	0.203	< 0.033	0.856	0.964	0.648	1.434
Smoking history	-0.047	0.271	0.031	0.861	0.954	0.561	1.623
ECOG PS	-0.166	0.274	0.365	0.546	0.847	0.459	1.450
Pathological type	1.587	0.492	10.393	0.001^*^	4.888	1.863	12.827
Differentiation	0.124	0.232	0.289	0.591	1.133	0.719	1.783
Number of brain metastasis	0.019	0.280	0.005	0.945	1.019	0.589	1.765
Radiotherapy			2.900	0.407			
Radiotherapy (1)	-0.108	0.248	0.188	0.664	0.898	0.552	1.460
Radiotherapy (2)	-0.245	0.351	0.486	0.486	0.783	0.393	1.558
Radiotherapy (3)	-0.704	0.423	2.779	0.095	0.494	0.216	1.132
EGFR			1.181	0.554			
EGFR status 1	-0.180	0.293	0.379	0.538	0. 835	0. 471	1. 482
EGFR status 2	0.035	0.301	0.014	0.907	1.036	0.574	1.868
EGFR-TKIs			2.610	0.271			
EGFR-TKIs (1)	0.012	0.236	0.003	0.959	1.012	0.637	1.609
EGFR-TKIs (2)	-0.350	0.240	2.131	0.144	0.705	0.441	1.127
Treatment line 2	-0.028	0.194	0.022	0.883	0.972	0.665	1.432
B: Partial regression coefficients; SE: Standard error; Exp: Exponential function. ^*^P < 0.05.							

### 2.4 OS影响因素分析结果

*Kaplan*-*Meier*法单因素生存分析结果显示，肿瘤分化程度是患者接受EGFR-TKIs后OS的影响因素[低分化*vs*其他，19.5个月（95%CI: 16.0-23.0）*vs* 32.4个月（95%CI: 26.1-38.7），*P*=0.002，图 2B]，而性别、年龄、吸烟史、病理组织类型、ECOG PS、脑转移病灶数、脑放疗情况、*EGFR*突变情况、EGFR-TKIs治疗时机等均不是患者接受EGFR-TKIs后OS的影响因素。OS的*Kaplan*-*Meier*法单因素生存分析结果详见表 4。

**4:** 总生存时间的*Kaplan*-*Meier*法单因素生存分析 Univariate survival analysis by *Kaplan*-*Meier* method for OS

Covariates	Comparisons	Median OS in months (95%CI)	*P*
Gender	Male *vs* Female	28.0 (16.9-39.1) *vs* 30.2 (22.2-38.1)	0.228
Age (yr)	≤60 *vs* > 60	32.4 (26.1-38.7) *vs* 27.2 (23.5-30.9)	0.163
Smoking history	None *vs* Yes	28.0 (22.8-33.3) *vs* 31.4 (15.7-47.1)	0.237
ECOG PS	0 *vs* 1	39.4 (17.8-57.1) *vs* 27.6 (22.3-32.9)	0.135
Differentiation	Low *vs* Other	19.5 (16.0-23.0) *vs* 32.4 (26.1-38.7)	0.002^*^
Pathological type	Adenocarcinoma *vs* Non-adenocarcinoma	29.1 (24.5-33.7) *vs* 15.3 (3.2-27.4)	0.125
Brain metastasis	Single *vs* multiple	31.4 (23.4-39.5) *vs* 28.0 (21.0-35.0)	0.577
Radiotherapy	No *vs* WBRT *vs* SRS *vs* SRS+WBRT	24.7 (15.2-34.2) *vs* 27.0 (19.5-34.5) *vs* 34.0 (22.1-45.9) *vs* 45.1 (19.4-70.7)	0.205
EGFR status 1	19del *vs* 21L858R *vs* Other	29.1 (23.6-34.7) *vs* 26.1 (13.3-38.8) *vs* 24.7 (12.2-37.2)	0.879
EGFR status 2	Common *vs* Other	28.0 (23.4-32.7) *vs* 24.7 (13.9-35.5)	0.292
EGFR-TKIs	Gefitinib *vs* Erlotinib *vs* Icotinib	30.2 (23.5-37.0) *vs* 32.0 (26.2-37.9) *vs* 23.6 (15.6-31.6)	0.099
Treatment line 1	First-line *vs* Second-line *vs* Other-line	30.2 (23.6-36.9) *vs* 28.0 (21.1-34.9) *vs* 28.0 (13.5-42.5)	0.823
Treatment line 2	First-line *vs* Non-first-line	30.2 (23.6 -36.9) *vs* 28.0 (20.7-35.4)	0.759
^*^P < 0.05.			

*Cox*多因素生存分析结果显示，肿瘤分化程度是患者接受EGFR-TKIs后OS的独立影响因素（*P*=0.050）。OS的*Cox*多因素生存分析结果详见表 5。

**5:** 总生存时间的*Cox*多因素生存分析 *Cox* multivariate survival analysis for OS

Covariates	B	SE	Wald	*P*	Exp (B)	95%CI for Exp (B)	
						Lower	Upper
Gender	0.065	0.306	0.045	0.833	1.067	0.586	1.942
Age	-0.265	0.261	1.030	0.310	0.767	0.460	1.280
Smoking history	-0.123	0.350	0.123	0.726	0.885	0.446	1.757
ECOG PS	-0.487	0.369	1.742	0.187	0.615	0.298	1.266
Pathological type	1.047	0.546	3.678	0.055	2.848	0.977	8.302
Differentiation	0.551	0.281	3.845	0.050^*^	1.735	1.000	3.010
Numbers of brain metastasis	0.092	0.324	0.080	0.777	1.096	0.581	2. 069
Radiotherapy			4.442	0.271			
Radiotherapy (1)	-0.247	0.315	0.612	0.434	0.781	0.421	1.450
Radiotherapy (2)	-0.586	0.452	1.677	0.195	0.557	0.229	1.351
Radiotherapy (3)	-1.126	0.594	3.591	0.058	0.324	0.101	1.039
EGFR			2.670	0.263			
EGFR status 1	-0.525	0.358	2.159	0.142	0.591	0.293	1.192
EGFR status 2	-0.196	0.370	0.282	0.596	0.822	0.398	1.697
EGFR-TKIs			5.985	0.050			
EGFR-TKIs (1)	0.383	0.315	1.476	0.224	1.466	0.791	2.718
EGFR-TKIs (2)	-0.452	0.304	2.200	0.138	0.637	0.351	1.156
Treatment line 2	0.148	0.251	0.345	0.557	1.159	0.708	1.897
^*^P < 0.05.							

### 2.5 亚组分析：存在19del和21L858R突变患者的临床特征和生存比较

存在常见突变（*EGFR* 19del和21L858R）的患者共119例，其中存在19del和21L858R的患者分别有68例和51例，其临床病理特征详见表 6。两组间各特征的差异均没有统计学差异。

**6:** 119例存在19del或21L858R患者的临床病理特征 Clinicopathologic characteristics of the patients harboring 19del or 21L858R (*n*=119)

Characteristic		EGFR 19del (*n*=68)	EGFR 21L858R (*n*=51)	*P*
		*n* (%)	*n* (%)	
Age (yr)	< 60	42 (61.8)	33 (64.7)	0.742
	≥60	26 (38.2)	18 (35.3)	
Gender	Male	31 (45.6)	24 (47.1)	0.873
	Female	37 (54.4)	27 (52.9)	
Smoking history	None	47 (69.1)	36 (70.6)	0.863
	Yes	21 (30.9)	15 (29.4)	
ECOG PS	0	12 (17.6)	7 (13.7)	0.563
	1	56 (82.4)	44 (86.3)	
Pathological type	Adenocarcinoma	5 (7.4)	3 (5.9)	1.000 (*Fisher’s* Exact test)
Non-adenocarcinoma	63 (92.6)	48 (94.1)		
Differentiation	Low	20 (29.4)	10 (19.6)	0.223
	Other	48 (70.6)	41 (80.4)	
Brain metastasis	Single site	24 (35.3)	12 (23.5)	0.167
Multiple sites	44 (64.7)	39 (76.5)		
Radiotherapy for Brain metastasis	No	17 (25.0)	9 (17.6)	0.816
	SRS	9 (13.2)	7 (13.7)	
	SRS+WBRT	6 (8.8)	5 (9.8)	
	WBRT	36 (52.9)	30 (58.8)	
EGFR-TKIs	Gefitinib	17 (25.0)	15 (29.4)	0.807
	Erlotinib	22 (32.4)	17 (33.3)	
	Icotinib	29 (42.6)	19 (37.3)	
Treatment line	First-line	43 (63.2)	35 (68.6)	0.540
	Other-line	25 (36.8)	16 (31.4)	

存在19del和21L858R患者的中位PFS分别是11.2个月（95%CI: 9.8-12.6）和8.9个月（95%CI: 7.2-10.7），两者间差异无统计学意义（*P*=0.546，图 3A）。存在19del和21L858R患者的中位OS分别是29.1个月（95%CI: 23.6-36.7）和26.1个月（95%CI: 13.3-38.3），两者差异无统计学意义（*P*=0.811，图 3B）。

**3:**
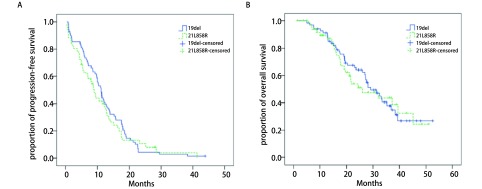
19del和21L858R患者的生存比较。A：19del和21L858R患者的无进展生存时间比较。存在19del和21L858R患者的中位PFS分别是11.2个月（95%CI: 9.8-12.6）和8.9个月（95%CI: 7.2-10.7），两者间差异无统计学意义（*P*=0.546）。B：19del和21L858R患者的总生存时间比较。存在19del和21L858R患者的中位OS分别是29.1个月（95%CI: 23.6-36.7）和26.1个月（95%CI: 13.3-38.3），两者差异无统计学意义（*P*=0.811）。 Comparisons of progression-free and overall survival between patients harboring EGFR 19del and 21L858R. A: Comparison of progressionfree survival between patients harboring EGFR 19del and 21L858R. The median progression-free survival for patients harboring EGFR 19del and 21L858R were 11.2 months (95%CI: 9.8-12.6) and 8.9 months (95%CI: 7.2-10.7), respectively. No statistical significance was observed between the two groups (*P*=0.546). B: Comparison of overall survival between patients harboring EGFR 19del and 21L858R. The median overall survival for patients harboring EGFR 19del and 21L858R were 29.1 months (95%CI: 23.6-36.7) and 26.1 months (95%CI: 13.3-38.3), respectively. No statistical significance was observed between the two groups (*P*=0.811).

## 3 讨论

本研究通过回顾性分析138例*EGFR*突变阳性合并脑转移的NSCLC的相关临床数据，发现该群患者接受一代EGFR-TKIs的疗效良好。此外，病理组织类型是患者接受EGFR-TKIs后PFS的影响因素及独立预测因素。而肿瘤分化程度是患者接受EGFR-TKIs后总生存时间的影响因素及独立预测因素。而19del合并脑转移和21L858R突变合并脑转移两亚组的患者在接受一线EGFR-TKI治疗时，虽有趋势表明19del的生存好于21L858R，但PFS及OS在两组间均未表现出统计学差异。

本研究入组女性患者居多，同时患者肺部原发病灶的病理组织学类型以腺癌居多，可能原因为肺腺癌在女性中发病率较高，女性患者*EGFR*突变率高，且腺癌血道转移多见且发生较早，故脑转移患者的病理类型以腺癌为主。本研究中入组的患者年龄以60岁以下为主。目前许多研究都发现了类似趋势。Bajard等^[13]^发现年龄≤62岁是发生脑转移的危险因素（HR: 2.5, 95%CI: 1.33-4.76; *P*=0.004）。Ceresoli等^[14]^认为年龄 < 60岁发生脑转移的概率更高（HR: 1.26, 95%CI: 1.03-1.53; *P*=0.03）。就本研究数据而言，一方面，随着我国肺癌发病率逐年上升，青年肺腺癌人数在总体肺腺癌中所占比例也呈明显上升的趋势。同时与一些生物学因素有关也有关系，例如年龄更小的患者，体内Ki-67水平更高，促进血管内皮生长因子（vascular endothelial growth factor, VEGF）的表达有关^[15, 16]^。因此，对于年龄更小的患者更容易发生脑转移的原因有待进一步的研究。

本研究结果显示，对于*EGFR*突变阳性合并脑转移的患者，接受一线EGFR-TKIs治疗时，组织病理学类型是无进展生存期的独立影响因素。其中腺癌患者的PFS要长于其他病理学亚型的患者。相关研究^[17]^提示，对于亚裔肺癌患者，接受吉非替尼作为二线及三线治疗时，腺癌患者的PFS较鳞癌患者明显延长。一个基于PubMed数据库开展的荟萃分析发现，对于*EGFR*突变的进展期非腺癌NSCLC患者接受吉非替尼治疗时，患者的有效率（response rate, RR）、疾病控制率（disease control rate, DCR）、mPFS比*EGFR*突变的腺癌患者要差，且其差异有统计学意义^[18]^。以上相关研究结果均证实，EGFRTKIs对于*EGFR*突变阳性的腺癌患者的疗效要好于非腺癌NSCLC，与本研究的结果一致。该差异可能与非腺癌患者EGFR-TKIs耐药发生率较高有关。EGFR-TKIs的耐药机制复杂，其中磷脂酰肌醇3-激酶/丝氨酸苏氨酸蛋白激酶/哺乳动物雷帕霉素靶蛋白信号转导通路（PI3K/AKT/mTOR）的活化是EGFR-TKIs耐药的常见原因之一^[19]^。研究*PIK3CA*基因突变与临床资料的相关统计学分析中发现，*PIK3CA*基因与病理类型显著正相关（*P* < 0.01），即鳞癌患者的*PIK3CA*基因突变率比腺癌高，更易发生耐药^[20-22]^。

本研究发现，肿瘤分化程度是*EGFR*突变合并脑转移患者接受EGFR-TKIs后OS的独立影响因素。首先，众所周知，高分化肿瘤的恶性程度相对较低，其预后一般相对较好。其次，肺癌的发生发展受多基因调控，从原位增殖性病变到侵袭、转移等一系列复杂的过程与抑癌基因*P53*突变（MtP53）、VEGF密切相关。有研究提示MtP53与肿瘤分化程度有关（*P* < 0.05）。MtP53阳性表达率在中低分化晚期NSCLC组织中明显升高，而且MtP53表达越高，患者生存率越低。提示MtP53在NSCLC发生和发展的过程中起重要调控作用^[23]^。此外，VEGF是一组生长因子，主要由肿瘤细胞产生，与肿瘤血管形成、侵袭、转移密切相关。研究^[23]^表明，VEGF的表达与肿瘤分化程有关（*P* < 0.05），分化程度越低，表达越强。而VEGF表达越高，生存时间越短。以上证据及本研究结果均表明，分化程度低的*EGFR*突变的NSCLC合并脑转移患者，接受一代EGFR治疗时，预后可能更差。

IPASS研究^[24]^奠定了EGFR-TKIs在晚期NSCLC患者中一线治疗的地位。目前指南也推荐将EGFR-TKI作为*EGFR*突变的NSCLC患者一线治疗手段，因为其RR、PFS、患者的生活质量均较一线使用细胞毒化疗药物的患者有明显改善^[25, 26]^。但也有研究^[27]^发现了不一样的结果，西班牙肺癌组织发现对于*EGFR*突变阳性的肺癌患者接受EGFR-TKIs治疗时，治疗时机对疗效没有影响。Koo等^[28]^在突变阳性的亚裔人群中也发现了EGFR-TKI治疗时机不是生存时间的独立影响因子。目前对NSCLC合并脑转移患者接受EGFR-TKIs治疗时机比较的研究不多，本研究发现*EGFR*突变阳性合并脑转移的患者，接受一代EGFR-TKIs治疗时，PFS是非一线治疗好于一线治疗，而OS是一线治疗好于非一线治疗。我们认为，导致这一结果的原因可能是分析PFS影响因素时存在混杂因素，也可能是目标人群不一样导致，故具体还有待进一步研究证实。

尽管*EGFR*突变阳性的患者接受EGFR-TKIs治疗疗效较好，但目前的一些研究发现，不同*EGFR*突变位点的患者接受EGFR-TKIs治疗时的效果是有差异的^[29-31]^。一些临床研究发现，相较于存在21L858R的患者，存在19del的患者PFS和OS更长^[27, 32, 33]^。然而，一项日本Ⅲ期临床试验发现不同*EGFR*突变状态的患者PFS之间并无明显差异^[34]^。本研究对于存在19del和21L858R突变患者的亚组分析发现，存在19del和21L858R患者在接受一代EGFRTKI治疗时，其中位PFS和中位OS间差异无统计学意义（*P*=0.811），但仅从生存曲线来看，有趋势表明19del好于21L858R。除考虑到研究不足和混杂因素外，还应注意到本研究全部患者均有脑转移，与以往研究的入组对象有差异。因此，脑转移患者中存在19del和21L858R的疗效及生存是否有差异，还有待进一步研究证实。

采用常规化疗及放疗治疗脑转移病灶副作用较大且疗效欠佳，靶向治疗的应用给晚期肺癌患者带来了新的选择。本研究初步分析了*EGFR*突变合并脑转移患者接受一代EGFR-TKIs后的生存情况及生存影响因素。发现病理组织类型及原发肿瘤的分化程度为突变阳性合并脑转移患者接受EGFR-TKIs治疗时预后的影响因素。但本研究仍然存在一些不足之处，首先，由于脑转移灶组织获取困难，故无法验证其与肺部病灶*EGFR*基因突变状况是否一致。其次，本研究是回顾性研究，资料的收集存在一定局限性，对于确诊脑转移的患者症状有无、脑转移病灶具体数目、体积和大小无法完全明确，故可能对于研究结果存在一定影响，同时不可避免的掺杂了一些没有考虑到的因素，引起一定的偏倚。还需要今后开展大样本量的前瞻性随机对照临床试验加以证实。
